# Reconstructing the household transmission of influenza in the suburbs of Tokyo based on clinical cases

**DOI:** 10.1186/s12976-021-00138-x

**Published:** 2021-02-10

**Authors:** Masaya M. Saito, Nobuo Hirotsu, Hiroka Hamada, Mio Takei, Keisuke Honda, Takamichi Baba, Takahiro Hasegawa, Yoshitake Kitanishi

**Affiliations:** 1 University of Nagasaki Siebold, Nagasaki, Japan; 2 The Institute of Statistical Mathematics, Tokyo, Japan; 3 Hirotsu Clinic, Kawasaki, Japan; 4 Shionogi & Co., Ltd., Osaka, Japan

**Keywords:** Influenza, Household transmission, Mathematical model, Stochastic simulation, Infectious period, Secondary attack ratio

## Abstract

**Background:**

Influenza is a public health issue that needs to be addressed strategically. The assessment of detailed infectious profiles is an important part of this effort. Household transmission data play a key role in estimating such profiles. We used diagnostic and questionnaire-based data on influenza patients at a Japanese clinic to estimate the detailed infectious period (as well as incubation period, symptomatic and infectious periods, and extended infectious period after recovery) and the secondary attack ratio (SAR) of influenza for households of various sizes based on a modified Cauchemez-type model.

**Results:**

The data were from enrolled patients with confirmed influenza who were treated at the Hirotsu Clinic (Kawasaki, Japan) with a neuraminidase inhibitor (NAI) during six northern hemisphere influenza seasons between 2010 and 2016. A total of 2342 outpatients, representing 1807 households, were included. For influenza type A, the average incubation period was 1.43 days (95% probability interval, 0.03–5.32 days). The estimated average symptomatic and infective period was 1.76 days (0.33–4.62 days); the extended infective period after recovery was 0.25 days. The estimated SAR rose from 20 to 32% as household size increased from 3 to 5. For influenza type B, the average incubation period, average symptomatic and infective period, and extended infective period were estimated as 1.66 days (0.21–4.61), 2.62 days (0.54–5.75) and 1.00 days, respectively. The SAR increased from 12 to 21% as household size increased from 3 to 5.

**Conclusion:**

All estimated periods of influenza type B were longer than the corresponding periods for type A. However, the SAR for type B was less than that for type A. These results may reflect Japanese demographics and treatment policy. Understanding the infectious profiles of influenza is necessary for assessing public health measures.

## Background

Simulation-based studies [1, 2] have been effectively used to assess the burden of influenza on society and the reaction of public health, as well as to help understand the dynamical nature of an epidemic [3]. The household is considered a particularly useful artificial experimental environment in which all members of the family are expected to experience intense contact [4, 5]. The risk of infection attributed to infective individuals is directly reflected in the data as a series of infection events in a particular household, which is largely independent of other households based on the intense contact. Accordingly, household data have played a key role in addressing estimation problems and have received particular attention in the analysis of influenza infection. In a number of previous studies, a Reed & Frost-type model has been used to estimate the probability that one susceptible subject will experience at least one contact with one infected household subject per unit time [6, 7] using a chain binomial model. In this type of model, the parameters are essentially estimated based on the number of infected subjects at each point in time. As an alternative, Longini et al. [8] proposed a constructive way to estimate the probability of being infected by an infective household member or community from the final numbers at the end of the epidemic. Carrat et al. [9] conducted a longitudinal study of the household transmission of influenza in the 1999/2000 season that included the start and end times of the illness for 946 households. Cauchemez et al. [4] applied a Bayesian Markov chain Monte Carlo (MCMC) to the outcome of the study to estimate the force of infection and the distribution of the infectious period simultaneously.

Although the infective period is one of the most clinically important parameters to describe natural history, it alone is not sufficient to describe influenza epidemics. There have been numerous attempts to identify other parameters, both in Japan and elsewhere. Frequency of social contacts and the secondary attack ratio (SAR), which is the probability of any member (of *n* - 1) being infected by the primary source, in household contacts are also essential factors for describing epidemics, in addition to the infectious and latent periods. Wallinga et al. [10] quantified the concept of social contacts in an age-specific contact matrix, which was then applied by Mossong et al. [11] in an investigation in Europe. Carcione et al. [12] estimated the SAR in households during the first circulation of the pandemic influenza A(H1N1) 2009. In Japan, Uchida et al. [13–15] conducted questionnaire-based studies of school outbreaks. Takeuchi et al. [16] and Ibuka et al. [17] investigated social contacts in a village in Miyazaki prefecture and among age-stratified responders recruited online, respectively. Nishiura & Oshitani [5] estimated the SAR in households for the pandemic influenza A(H1N1) 2009.

Hirotsu et al. [6] used data from the Hirotsu Clinic in Kawasaki City, a major city in the greater Tokyo area, to conduct a single-center, prospective, observational study (UMIN-CTR: UMIN000024650) involving the transmission of influenza during six influenza seasons (2010–2016). The data were taken from the records of 2342 outpatients, representing 1807 households, who were diagnosed with influenza A or B. Each household record consisted of the diagnosis as well as the infection history of other household members who were tracked via a questionnaire provided to the outpatients. These records serve as the basis for the current study.

Given the underlying information available in the diagnostic records of the Hirotsu Clinic, we sought to estimate the latent and infectious periods and to reconstruct household transmission in a pair of simulations. The latent period of influenza is hardly identifiable via routinely corrected epidemic data except when a small outbreak occurs, one induced by clearly identified primary cases [6]. Moreover, asymptomatic agents may have a non-negligible influence on the epidemic [18, 19].

We employ a modified Cauchemez-type household transmission model. After conducting two simulation trials to produce a detailed infection profile, we explore the information extracted from the Hirotsu Clinic’s influenza diagnosis records. In the first step, estimates of the infectious period are produced by combining the available records and the simulation model. In the second step, we simulated inter-household transmission assuming a particular force of infection between households and calibrate the assumed value so that the number of simulated infected households for each household size agrees with the reality represented in the data. It is expected that the new and more detailed infectious profile developed here for influenza will contribute to public health globally.

The remainder of the paper is organized as follows: In the Methods section, the dataset is introduced and the methods applied to the dataset are described mathematically. In the Results section, parameter estimates related to the natural history of influenza are presented. In the Discussion section, we summarize the outcomes of our trials and discuss the study’s strengths and limitations. Finally, we offer concluding remarks and indicate future research directions in the Conclusion section.

## Methods

### Data source

**Fig. 1:**
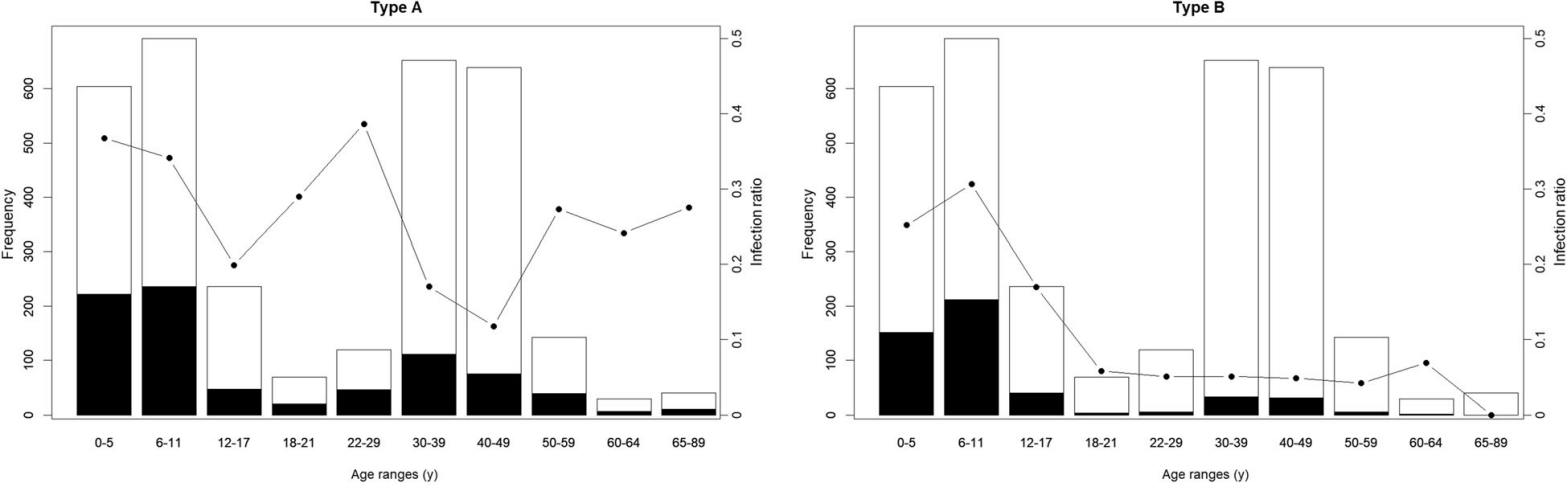
Age-specific distributions of the total infected cases over six years (black portion of the histograms) and the corresponding age-specific population (black+white) for influenza A and B. The lines show a crude estimate of the infection probability, calculated as the number of infectious individuals divided by the population

The data were derived from enrolled patients with confirmed influenza who were treated at the Hirotsu Clinic (Kawasaki, Japan) with a neuraminidase inhibitor (NAI) during the six northern hemisphere influenza seasons between 2010 and 2016. A total of 2342 outpatients, representing 1807 households, were included (Table 1). A majority of the households have 3–5 members (parents and their children), as summarized in Table 1b. This is reflected in the age-specific distribution of the total infected cases over the six-year period shown in Fig. 1, where the 0–11 age group (preschool and elementary school children) and the 30–49 age group are predominant. The dataset includes individuals based on infection, either diagnosed or indicated by the family. For this reason, the corresponding ‘divisor’ necessary to obtain a crude estimate of the infection probability is not clearly defined. The divisors are calculated by counting all members of the household once a year if the household experiences the disease. The jagged lines in Fig. 1 show the crude infection probability for influenza A or B. While a general downward trend (see Ref [20]) is apparent in the case of type B, there is no clear trend for type A. Patients of any age who were diagnosed with influenza A or B using rapid influenza diagnostic tests (RIDTs) were eligible for inclusion in the study. ImmunoAce® Flu (Tauns Laboratories, Inc., Shizuoka, Japan) was used for the differential diagnosis of influenza A and B. In order to share the data among members of the research team, the original Hirotsu Clinic data were first anonymized by keeping only assigned identifiers, along with the times of infection-related events and the relationship between identifiers in order to reconstruct households. This anonymization did not affect the analysis. The infection events consisted of the onset (based on questionnaire responses), diagnosis at the clinic, and recovery (occasionally N/A was entered to indicate the disappearance of constitutional symptoms, specifically antipyresis < 37.5 °C). The dosage and administration of the NAIs were as per the package insert for each product. Secondary infection patients were defined as household members who were diagnosed with the same influenza type as the index patient within 8 days after the onset of symptoms in the index patient. Occasionally, two or more members of a household may be simultaneously (or nearly so) infected outside the household and be introduced into the household as primaries. Figure 1 shows that the serial interval frequency decreases monotonically up to 0.5 days for influenza type A and up to 1.25 days for influenza type B. This downward trend may be at least partially attributable to the introduction of multiple primaries, as discussed in more detail later.

**Table 1:** Summary of diagnosed influenza cases covered by our questionnaire investigation

Year	Type		Cases		Households		Total Cases	
a. Number of cases in each year								
1	A		243		189			
	B		104		86			
2	A		263		204			
	B		222		177			
3	A		259		189			
	B		11		8		270	
4	A		175		147			
	B		289		227		464	
5	A		339		249			
	B		18		13		357	
6	A		237		168			
	B		182		150		419	
	Total		2342		1807			
b Number of households of each household size								
**Household size**	1	2	3	4	5	6	7	**Total**
**A**	37	62	318	544	149	30	6	1146
**B**	16	15	182	340	85	18	5	661
**Total**	53	77	500	884	234	48	11	1807

### Transmission model in a household

**Fig. 2:**
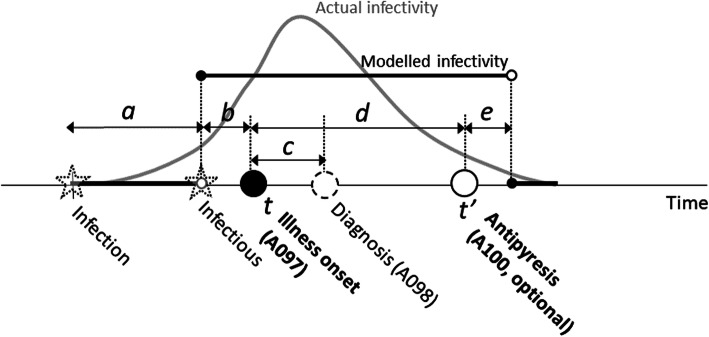
Natural history of infective people and the variation of infectivity. In an actual situation, a person may be infected at some unknown point in time (Infection) and the infectivity to other people gradually increases up to its maximum around the time when the illness is well developed and recognized (Illness onset). It then is drained as the process of recovery from infection proceeds, which may be clinically observed by antipyresis (Antipyresis), though weak infectivity may remain. For simplicity, such time variation of infectivity is modeled using a piecewise constant function that takes a non-zero constant value *λ*_0_ only from one point in time (labeled as Infectious) to another point near Antipyresis. The modeled infectivity function is temporally controlled by four period parameters: *a* (pre-symptomatic and non-infectious), *b* (pre-symptomatic and infectious), *d* (symptomatic and infectious) and *e* (extended infective after recovery)

Cauchemez et al. [4] conducted a longitudinal investigation of 334 households over a 15-day period in winter 1999–2000 and applied their novel household transmission model to estimate parameters describing the natural history of (seasonal) influenza infection. The approach we propose is similar to this in concept. In the model, the time variation of the infectivity attributed to an individual is modeled as a piecewise constant function (Fig. 2) that includes four period parameters: pre-symptomatic and non-infective (*a*), pre-symptomatic and infective (*b*), symptomatic and infective (*d*), and extended infective after recovery (*e*). By definition, infectivity sustains for period *b* + *d* + *e*. Period *d* was observed from the data; period *b* + *e*, on the other hand, was estimated via maximum likelihood estimation. Because the individual values of *b* and *e* could not be broken out from the combined *b* + *e* value, we set *b* = 0 only for conceptual completeness.

We employ a parametric model (which will be explained in the next subsection) to describe *a* and *d* as random variables and obtain their point estimates via maximum likelihood estimation. The subscripted version (e.g., *a*_*i*_ for *a*) denotes the values for an individual *i*. We assume that a given individual may be infected per unit time according to the probability of the sum of the infectivity attributed to the rest of the family members. Specifically, if family member *i* acquires infectivity at time *t*_*i*_ – *b*_*i*_ (*t*_*i*_ is the time of the illness onset informed by the data) and loses it at ${t}_i^{\prime }+{e}_i$ (${t}_i^{\prime }$ is the time of the disappearance of constitutional symptoms, which may be unavailable in the data), then the infection probability per unit time (i.e., the force of infection, FOI) that member *j* is infected at time *t* is given by

1 $$ {\lambda}_j(t)=\frac{\lambda_0}{{\left(n-1\right)}^{\alpha }}{\sum}_{i\ne j}1\kern1px 1\kern0.28em \left({t}_i-{b}_i\le t\le {t}_i^{\prime }+{e}_i\right), $$

where *n* is the number of members in the household (i.e., the household size), *λ*_0_ is a constant controlling the FOI, and $1\kern1px 1\kern0.28em \left(\cdot \right)$ is an indicator function: $1\kern1px 1\left(\mathrm{True}\right)=1\kern0.28em \mathrm{and}\kern0.28em 1\kern1px 1\left(\mathrm{False}\right)=0$. Cauchemez et al. [4] employed this type of power-law risk (assuming *λ*_*j*_(*t*) ∝ *n*^−*a*^) to check the effect of the density of infectives and obtained an estimate of *α* = 0.84 (95% CrI: 0.46–1.21). Later, Ferguson et al. used *α* = 0.8, a value close to the mean determined by Cauchemez et al., in their pandemic simulation study [1]. Division by the number of other family members (the case of *α* = 1) assumes that infective contacts occur in an exclusive time-sharing manner; the absence of the division (*α* = 0) implies that the FOIs exert influence equally on the population of concern, irrespective of family size. The former setting is appropriate for diseases that require close contact for infection, including influenza, while the latter well matches, for example, diseases where the infection is induced by polluted agents [21]. However, as we will see, because the SAR is non-negligibly large in large families, setting *α* = 1 appears to over-reduce the FOI: an infective agent may have a conversation with two or more family members. For this reason, we introduced an empirically determined power for scaling.

### Estimation of parameters

**Fig. 3:**
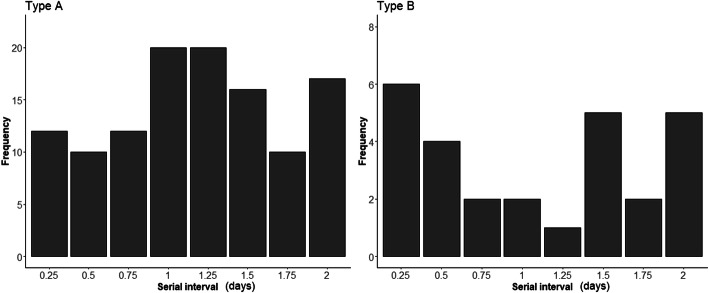
Partial serial interval histograms of the crude number of secondary cases. Only the left side of the histograms (≤ 2 days) is shown. In the type A histogram, there is a downward trend up to 0.5 days; in the type B histogram, there is a downward trend up to 1.25 days (type B). These may be attributable to simultaneous infections outside the household

**Fig. 4:**
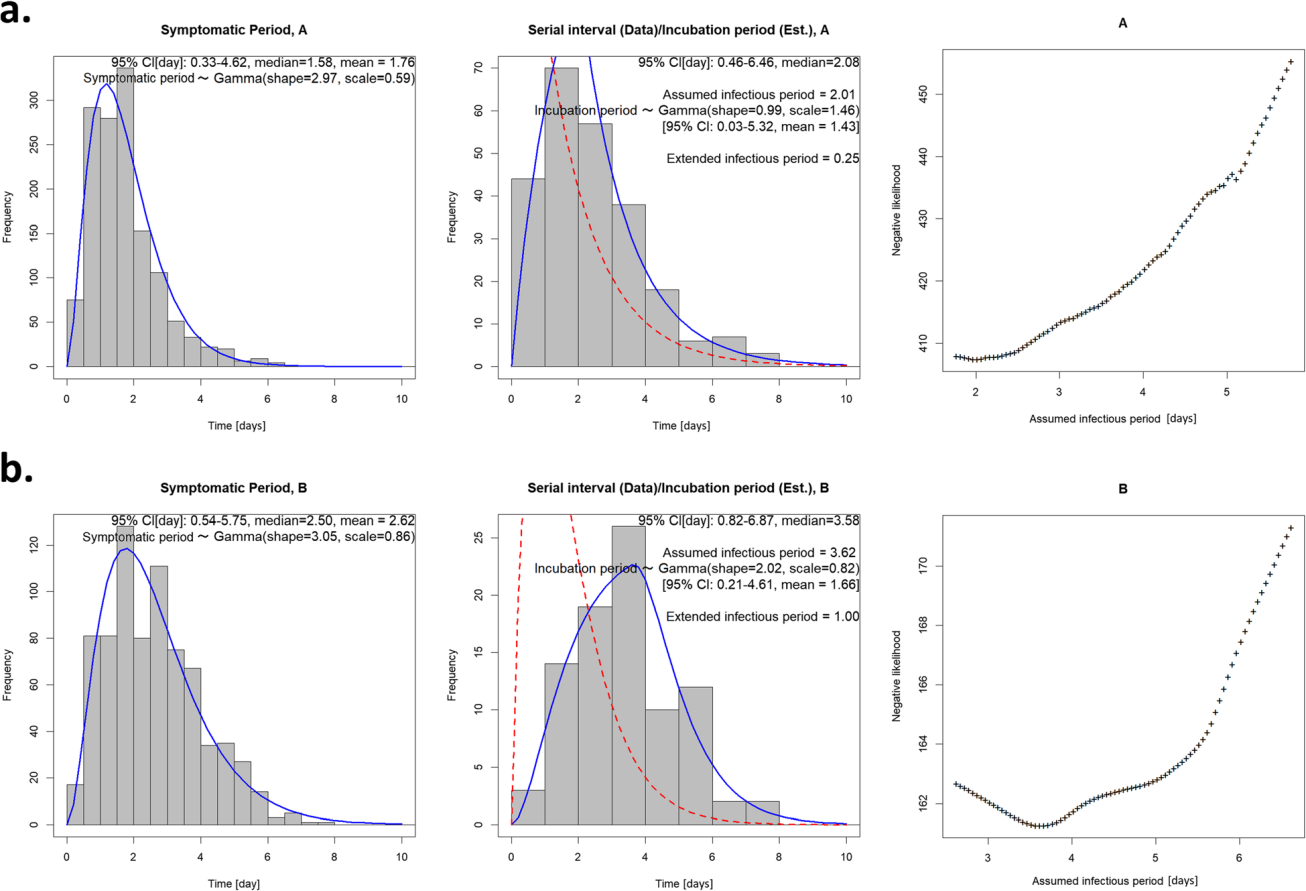
Summary of household infection data and estimates of the parameters for influenza types. **a**. Type A and **b**. type B (bottom row). The left column shows the histogram of the symptomatic period and its fitting to a gamma distribution. The middle column shows the histogram of the serial interval, its fitting to the distribution constructed as a convoluted gamma distribution (blue curve), and the extracted infectious period (annotation) and incubation period (red curve) via the fitting (see the Methods section for detail). The right column shows the negative likelihood against the assumed infective period

We employ a rather descriptive statistical approach to estimating *a*, *d,* and *e*. Setting *b* = 0, the symptomatic period *d*^(*h*, *i*)^ of the *i*-th infected member in household *h* is informed by data as *t*^′(*i*)^ − *t*^(*i*)^; summing such realizations over all households and members therein, we have the empirical distribution (i.e., histogram) of period *d*, along with its mean E[*d*]. Similarly, in principle, collecting the serial interval instance ${t}_{\mathrm{int}}^{(h)}:= {t}^{\left(h,2\right)}-{t}^{\left(h,1\right)}$ over households *h*, we have the empirical distribution for *t*_int_. As mentioned in the data source subsection, however, cases in which two family members who were simultaneously infected (or nearly so) outside the home may occasionally appear. In the expert opinion of one of the authors, an infected agent barely develops sufficient infectiousness within 24 h. In our study, a serial interval within 24 h was observed in 54 of 290 influenza A pairs (18%) and in 14 of 135 influenza B pairs (18%). It may be that these simultaneous pairs increased the proportion of short serial intervals in the distribution. Fig. 3 shows the left ends of the histograms of the crude distributions of *t*_int_ for influenza A and B, respectively. As is apparent in figure, for influenza B, there is a persistent downward trend up to 1.25 days, followed by an upward trend that forms the left side of an approximately bell-shaped distribution (the full histogram is shown in Fig. 4). Though there is a possibility that a relatively large infectious incubation period of a primary can yield a small (occasionally negative) serial interval, our dataset is not sufficient to deal separately with the infectious incubation period (as noted above). As a consequence, we empirically adjusted the contribution from simultaneous primary cases in the crude serial interval distribution under the assumption that this downward trend is mainly attributed to simultaneous infections, in particular where *t*_int_ is close to zero. Given that this trend is almost linear, we assumed that the count between *t*_int_ and *t*_int_ + Δ*t* is attributable to true household transmission with probability ∝*t*_int_Δ*t* and discarded stochastically the data of *t*_int_ < 1.25 days accordingly. The same procedure was applied to influenza A, with a different cut-off of *t*_int_ < 0.5 days, although the downward trend here is not as apparent as in the case of influenza B. After these adjustments, the “observed” serial interval *t*_int_ can be modeled as the summation of the interval *τ*_int_ between the infection times of the primary and secondary infections (i.e., the “intrinsic” serial interval) and the incubation period *a* of the secondary subject. For ease of computation of the *a* distribution, we introduce two simplifications. First, the interval of the two cases is assumed to follow a uniform distribution truncated at the mean infective period *τ*_ifv_: $p\left({\tau}_{\mathrm{int}}\right)=1\kern1px 1\kern0.28em \left(0\le {\tau}_{\mathrm{int}}\le {\tau}_{\mathrm{ifv}}\right)/{\tau}_{\mathrm{ifv}}$ .  Second, the incubation period follows a gamma distribution: *a* ∼ Gam (shape = *k*_*a*_, scale = *θ*_*a*_). The distribution form of *t*_int_ is then written as

$$ {\displaystyle \begin{array}{rcl}p\left(\operatorname{}{t}_{\mathrm{int}}|{k}_a,{\theta}_a,{\tau}_{\mathrm{ifv}}\right)& =& \frac{d}{{d t}_{\mathrm{int}}}{\int}_0^{\min \left({t}_{\mathrm{int}},{\tau}_{\mathrm{ifv}}\right)}\frac{1}{\tau_{\mathrm{ifv}}}{\int}_{\tau_{\mathrm{int}}}^{t_{\mathrm{int}}}\mathrm{Gam}\left({t}_{\mathrm{int}}^{\prime }-{\tau}_{\mathrm{int}}|{k}_a,{\theta}_a\right){d t}_{\mathrm{int}}^{\prime }{d\tau}_{\mathrm{int}}\\ {}& =& \frac{1}{\tau_{\mathrm{ifv}}}{\int}_0^{\min \left({t}_{\mathrm{int}},{\tau}_{\mathrm{ifv}}\right)}\mathrm{Gam}\left({t}_{\mathrm{int}}-\tau |{k}_a,{\theta}_a\operatorname{}\right) d\tau \end{array}} $$

By maximizing the likelihood ${\prod}_hp\left({t}_{\mathrm{int}}={t}_{\mathrm{int}}^{(h)}|\ {k}_a,{\theta}_a,{\tau}_{\mathrm{ifv}}\right)$, we have the distribution of incubation period *a*. Technically, the simultaneous optimization of (*k*_*a*_, *θ*_*a*_, *τ*_ifv_) is sensitive to the initial conditions. Hence, given *τ*_ifv_ in a certain range, we optimize for (*k*_*a*_, *θ*_*a*_). Since a point estimate of the infective period *τ*_ifv_ can be equated to E [*d* + *e*], *τ*_ifv_ − E[*d*] serves as a point estimate of *e*. Scaling power *α* is determined as follows. The SAR is $1-{e}^{-\lambda {\tau}_{\mathrm{ifv}}}\approx \lambda {\tau}_{\mathrm{ifv}}$ with *λ* = (*n* − 1) · *λ*_0_/(*n* − 1)^*α*^, from Eq. (1). Our dataset allows us to compute the SAR for household sizes *n* = 3, 4, and 5 which together comprise approximately 90% of all households (a small number of households were of size 6 and 7). Therefore, the value of *α* with *λ*_0_*τ*_ifv_ is determined by the regression

$$ \log \mathrm{SA}{\mathrm{R}}_n=\log {\lambda}_0{\tau}_{\mathrm{ifv}}+\left(1-\alpha \right)\log \left(n-1\right). $$

The value of *λ*_0_ is determined as an MLE via a comparison of the number of secondary cases in the data and in the simulation. Suppose that *i* secondary cases appear in a simulated household of size *n* with probability *p*_*i*/*n*_ and that ${\left({p}_{i/n}\right)}_{i=0}^{n-1}$ is obtained through multiple simulation runs with different seeds. Then the likelihood for the comparison is $L\left({\lambda}_0\right)={\prod}_{n=3,4,5}{\prod}_{i=0}^{n-1}{\left({p}_{i/n}\right)}^{m_{i/n}}$, given *m*_*i*/*n*_ real households yielded *i* secondary cases. In other words, *λ*_0_ is chosen so that the KL divergence is minimized.

## Results

### Estimation of duration parameters

To estimate the duration parameters, we first summarized the dataset in the form of histograms for the symptomatic period and the serial interval. The dataset histograms for influenza A, along with the related estimation results, are shown in Fig. 4a. We identified 1389 cases in which the patient exhibited symptoms and 290 transmissions from the primary to the secondary subject. The symptomatic period distribution is well approximated by a gamma distribution with shape = 2.97 and scale = 0.59 days; that is, period *d* is 1.76 days on average, with a 95% probability interval (hereafter 95% I) of 0.33–4.62. The point estimate of the infective period as the MLE is 2.01 (left-panel in Fig. 4a). The difference between the infective and symptomatic periods is the extended infective period after recovery, *e*; here, *e* = 2.01–1.76 = 0.25 days. The incubation period is extracted as a gamma distribution with shape = 0.99 and scale = 1.46 days (mean: 1.43 days; 95% I: 0.03–5.32 days).

The results for influenza B are shown in Fig. 4. For the analysis here, we identified 760 cases where the patient exhibited symptoms and 135 transmissions from the primary to the secondary subject. A gamma distribution with shape = 3.05 and scale = 0.86 days (mean: 2.62 days; 95% I: 0.54–5.75 days) was fitted to the symptomatic period data; notably, the period here is longer than in the case of influenza A. The serial interval was also longer and cut off at approximately 6 days, while the infective period was estimated to be 3.62 days, which yields an incubation period of 1.66 days (95% I: 0.21–4.61 days), as well as a relatively long extended infective period after recovery, *e* = 3.62–2.62 = 1.00 days.

### Estimation of FOI

**Fig. 5:**
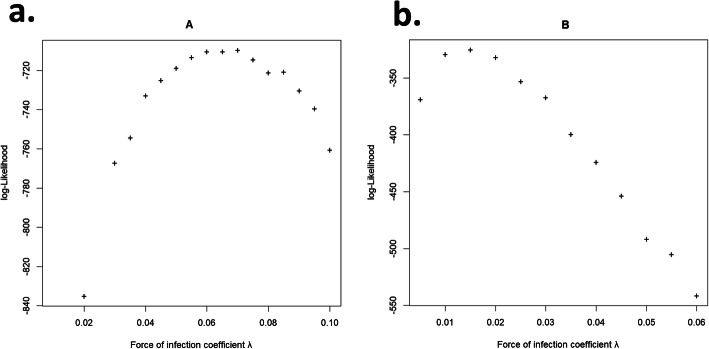
Likelihood curves for the two influenza types. **a**. Type A and **b**. type B are shown with respect to the number of secondary cases in a household as a function of the force of infection (FOI) coefficient *λ*_0_. The likelihood values are determined by simulation runs with different values of *λ*_0_ and Δ*λ*_0_ = 0.005; optimal values of *λ*_0_ are 0.065 for A and 0.015 for B

**Fig. 6:**
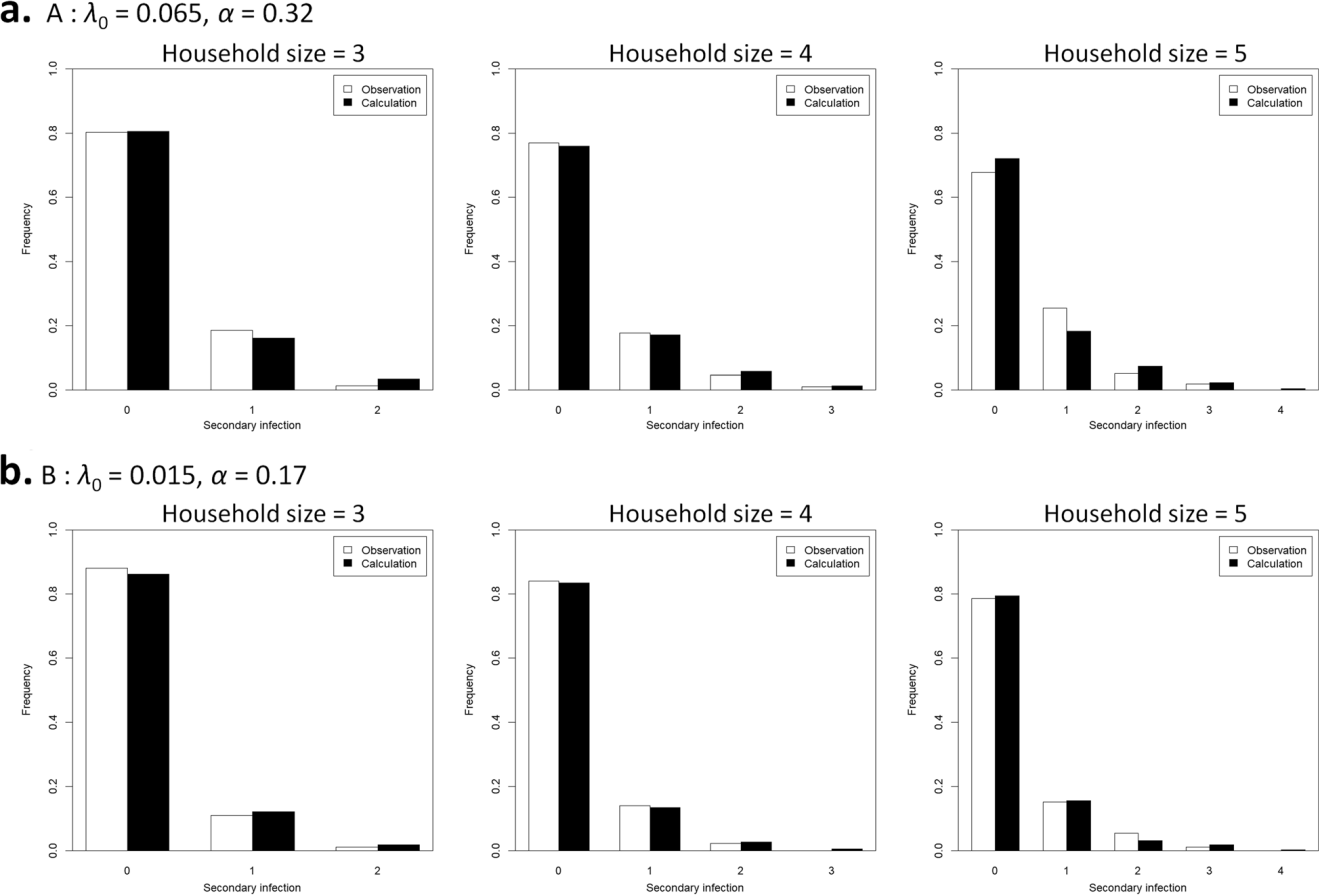
Comparison of the number of secondary cases in a household. Households have 3, 4, or 5 members in the actual data (white) and the simulation (black) for the two influenza types: **a**. type A and **b**. type B. The simulation run is mainly controlled by the force of infection (FOI) coefficient *λ*_0_ and the household size scaling power *a*; their ML estimates are shown

We then produced a point estimate of the FOI coefficient *λ*_0_ after fixing scaling power *α*. In the case of influenza A, the SAR increased from 20 to 32% as household size increased from 3 to 5, for which *α* = 0.32 is optimal. This is a much smaller value than that used in a previous study, where Ferguson et al. [1], who carried out agent simulations and employed a Cauchemez-type model as an internal process, used *α* = 0.8. With *α* = 0.32 and an infective period of 3.73 days, we ran 1024 simulations to obtain the ML estimate of *λ*_0_. The likelihood as a function of *λ*_0_ is shown in Fig. 5a; the optimal value was found to be *λ*_0_ = 0.065. The corresponding SAR distribution is shown in Fig. 6a. As shown, the simulated distribution well reproduces the data.

The same procedure was applied to influenza B. Compared to type A, type B exhibited a smaller SAR (range: 12–21%) and a much longer infective period (5.73 days). The resulting estimates are also different: *α* = 0.17 and *λ*_0_ = 0.015. The likelihood function is shown in Fig. 5b. The SAR distribution in Fig. 6b is similarly reproduced, and two or more secondary cases appear less frequently than for type A.

## Discussion

We estimated parameters to describe an influenza natural history, the incubation and infective periods, and the FOI coefficient, using both diagnostic and questionnaire-based data obtained in a clinic located in the suburbs of Tokyo. While the study produced several useful insights, it is not without limitations. Although the estimated incubation period is rather inescapably obscure because it is hidden by the different symptomatic periods for individuals and was reflected in a quite skewed distribution with shape less than unity, it was estimated to be roughly 1.5 days, both for type A and type B. For the latter, a uniform infective period distribution with bi-level infectivity (non-infective or infective) does not fully capture the nature of the influenza (e.g., virus titer over time). The difference between the infective period and the symptomatic period, for which we produced point estimates, was 0.25 days for influenza type A and 1.00 days for type B. While the estimate for type B would seem to be consistent with what is commonly known about this disease, the estimate for type A appears excessively short. According to the Enforcement Regulations for School Health and Safety Act in Japan [22], patients with an influenza virus infection are banned from attending school for 2 days (or 3 days for infant children) after the resolution of fever. Our estimates of the mean of the extended infective period after the resolution of fever were less than 2 or 3 days and were thus consistent with the enforcement regulation. In this respect, our constructed models would appear to be realistic. However, it should be noted that the choice of cut-off points to split simultaneous infection from true household transmission is influential in the estimation and, although the choice in the case of type B is rather clear, it is less obvious for type A.

**Fig. 7:**
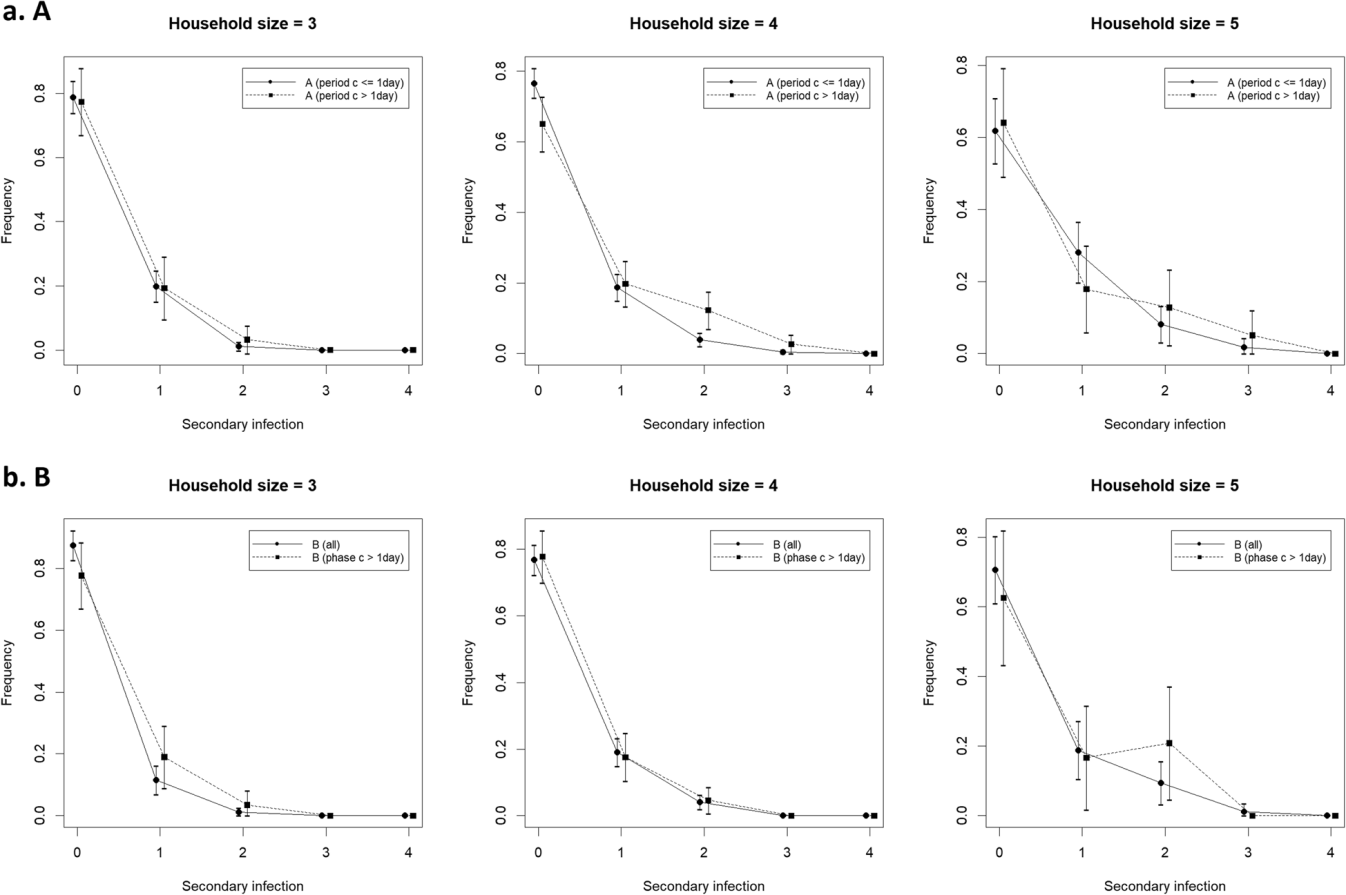
Comparison of the number of secondary cases in households with *c*_1_ ≤ 1 (*early*-diagnosed primary) and those with *c*_1_ > 1 (*late-*diagnosed primary): **a**. type A and **b**. type B

The scaling power was estimated to be quite small (*α* = 0.2 to 0.3) relative to that reported by Cauchemez et al. However, it should be noted that Cauchemez’s *α* = 0.84 estimate involved substantial uncertainty (95% CrI: 0.46–1.21), and that the best fit power is different for 1/*n*^*α*^ versus 1/(*n* − 1)^*α*^ (for example, 1/(*n* − 1)^0.3^ well fits to 1/*n*^0.42^). A straightforward explanation for this might be that the dataset covered households in which family members tended to spend most of their time with one another. However, two (or more) primary cases introduced almost simultaneously can elevate the apparent SAR in large families. Our analysis assumed that the first reported case infected the second, and so on. The fraction of the ignored tail (> 8 days) should be available to model the probability that multiple members were infected simultaneously, referring to epidemic surveillance in Tokyo. It should also be noted that the SAR may be underestimated due to vaccination and the basic strategy of early diagnosis followed by early treatment. While our dataset does not inform the vaccine effect separately since vaccination records are available only for infected household members, we can compare the SAR values between all households and those with a late-diagnosed primary case. Here, we identify a primary to be late-diagnosed if the waiting time *c* from the onset to the diagnosis is greater than 1 day, which is the case for 25% of households with type A and 33% of those with type B. Figure 7 shows the number of secondary cases according to household size. The uncertainties at the various points on the distributions are 95% CIs based on binomial sampling. Considering only the mean (crude proportion), the weights shift to a larger number of secondary cases, particularly for type A in four-member households. Late diagnosis and reduced awareness may raise the probability of household outbreaks. However, the uncertainties of the stratified and unstratified distributions overlap one another and we did not find a significant difference.

## Conclusion

As this study was conducted in Japan, the results are likely to reflect Japanese demographics and treatment policy. Although it is important to assess infectious profiles in various countries, the number of studies that have estimated the infectious duration and FOI of influenza in Japan is quite limited. In general, it is useful to understand the infectious profiles of influenza for examining public health measures.

We expect that our estimates of the infective period based on the present situation in an urban area in Japan will be informative to school health officials and helpful in determining the span of school closures and attendance suspensions. However, more research will be needed to improve the accuracy and applicability of our results.

## Data Availability

Not applicable.
